# Effectiveness of Visual-Tactile Examination and DIAGNOdent Pen in Detecting Early Enamel Caries and Its Remineralisation: An In Vitro Study

**DOI:** 10.1155/2022/1263750

**Published:** 2022-01-11

**Authors:** Mohammed Fadhil Rashid, Mohmed Isaqali Karobari, Mohamad Syahrizal Halim, Tahir Yusuf Noorani

**Affiliations:** ^1^Conservative Dentistry Unit, School of Dental Sciences, Universiti Sains Malaysia, Health Campus, Kubang Kerian, Kota Bharu, 16150 Kelantan, Malaysia; ^2^Department of Restorative Dentistry & Endodontics, Faculty of Dentistry, University of Puthisastra, Phnom Penh 12211, Cambodia; ^3^Center for Transdisciplinary Research (CFTR), Saveetha Dental College, Saveetha Institute of Medical and Technical Sciences, Saveetha University, Chennai, Tamil Nadu 600077, India; ^4^Conservative Dentistry Unit, Hospital Universiti Sains Malaysia, Health Campus, Kubang Kerian, Kota Bharu 16150, Kelantan, Malaysia

## Abstract

**Background:**

The caries preventive effect of Colgate Duraphat® and GC Tooth Mousse Plus® has been widely studied, but the remineralisation potential of initial occlusal caries using these two remineralisation materials remains unclear.

**Aim:**

This study is aimed at evaluating and comparing the remineralisation of early enamel caries on the occlusal surface of permanent posterior teeth using ICDAS II caries scoring system and DIAGNOdent Pen (DDPen) after remineralisation with Colgate Duraphat® and GC Tooth Mousse Plus®.

**Materials and Methods:**

Extracted posterior teeth (*N* = 120) with incipient occlusal caries were included in this study. The occlusal surface of each tooth was scored using DDPen and ICDAS II scoring before remineralisation. Then, remineralisation of the teeth of the experimental group was carried out using either CPP-ACP-F or fluoride varnish. After the remineralisation procedures, the occlusal surface of each tooth was again scored using DDPen and ICDAS II scoring. The teeth were then fixed in dental stone blocks and sectioned longitudinally for histological examination using a stereomicroscope. Statistical analysis was performed to calculate the sensitivity and specificity of DDPen and ICDAS II to detect remineralisation and compare with the gold standard histological examination.

**Results:**

According to ICDAS-II scores, a significant difference was noted in GC Tooth Mousse Plus® and Duraphat® study samples, whereas the difference between the pre-and post-remineralisation of the control group was not significant. According to the DDPen score criteria, a statistically significant difference was noted among all study groups; however, a greater significance level was noted in the GC Tooth Mousse Plus® and Duraphat® study samples compared with the control group. The Spearman's rank correlation of ICDAS-II and DDPen with Downer's histological score (gold standard) revealed a higher association of DDPen score (.738) as compared to ICDAS-II scores (.430).

**Conclusion:**

The study concluded that both ICDAS II and DDPen could detect remineralisation of early enamel occlusal caries. DDPen was more sensitive than ICDAS-II to detect remineralisation compared with the Downers histological scores.

## 1. Introduction

Dental caries is a gradually progressive disease that has been identified as the most commonly occurring oral disease/chronic infection [1, 2]. The diagnosis of a noncavitated carious lesion is considered challenging because this lesion can be disguised by the remineralisation effect of fluorides [3]. Visuo-tactile inspection of the carious lesion is an inexpensive and the most utilised method of caries assessment [4]. The accuracy and reliability of a diagnosis depend on the expertise and training of the assessor. A standardised caries assessment system is of paramount importance [5].

Several studies have reported that Duraphat® is highly effective in remineralising enamel from other mineralisation products [6–9]. Further research has found that a high concentration of fluoride treatments in acidic pH is more effective in reshaping the surface of noncavitated caries and encouraging fluoride absorption from those in neutral pH [10]. Casein phosphopeptide-amorphous calcium phosphate has been highlighted to enhance the stability of high calcium levels and enhance the delivery of ions to the tooth surface [11, 12]. A clinical trial involving CPP-ACP containing gum demonstrated decelerated progression and enhanced regression of carious lesions [13]. CPP-ACP application also showed postorthodontic white spot regression [14]. Furthermore, CPP-ACP has a significant remineralisation effect, and it can be considered a salivary biomimetic, as it shares many similarities with statherin [15].

ICDAS II supplies a consistent method of lesion detection and evaluation, leading to the diagnosis of caries [16]. ICDAS II provides good reproducibility and accurate detection in vivo and in vitro for initial caries lesions at different stages. The laser fluorescence method (DIAGNOdent) assists the detection of occlusal and approximal caries [2]. The use of the DDPen on white spot carious lesions [17] and an enamel window at the buccal surface in the middle one-third of the crown [18] has been reported. However, the use of DDPen to assess the remineralisation of the fissures, pits, and smooth surfaces has not been explored. Previous studies employed a relatively smaller sample size in their studies [19, 20]. Varying cut-off values have been used and recommended by previous studies [21, 22]. However, these values are dependent on various factors which include type of surface (smooth or fissure), physical properties (demineralisation or remineralisation), and the extent of the lesion (lesion confined to enamel only and lesion involving enamel and dentin).

Narrative and systematic reviews have shown that DDpen is more sensitive to the occlusal aspect of posterior teeth than traditional diagnostic methods, but the specificity is inferior to clinical visual examination [23–25]. DDPen also tends to overestimate deeper caries or dark (stained) lesions. Bhat et al. [26] conducted a systematic review assessing different cut-off values and concluded that the cut-off values based on the laboratory studies were not clinically correlated. The study suggested that the cut-off values may be selected based on the extent of the carious lesion, and different cut-off values were correlated with enamel and dentinal carious lesions.

Different cut-off values for DDPen have been described in the literature; however, no study has evaluated pre-and post-remineralisation relevance of cut-off values. Moreover, no previous studies have been carried out performing simultaneous comparison between the three assessment tools (ICDAS II, DDPen, and histological examination) on tooth surface remineralisation. Hence, this study is aimed at investigating the potential of ICDAS II and DDPen in detecting remineralisation of the early carious lesion after applying topical fluoride gel (Colgate Duraphat®) and casein phosphopeptide-amorphous calcium phosphate fluoride (Tooth Mouse Plus) at the occlusal surface of extracted human permanent posterior teeth. This study also compared the diagnostic cut-off values of DDPen corresponding with the histological examination after the remineralisation process.

## 2. Materials and Methods

Ethical approval for the current study was obtained from the Human Research Ethics Committee of Universiti Sains Malaysia (USM) (USM/JEPeM/18100516). Extracted permanent maxillary and mandibular posterior teeth for orthodontic, periodontal, or other reasons from adult patients that visited the outpatient dental clinics of the School of Dental Sciences, USM, were selected for this study.

A total of 120 freshly extracted teeth were included in this study. Before caries assessment, the occlusal surfaces were cleaned using a toothbrush with pumice slurry and water before randomly assigning numbers from 1 to 120. Unrestored mandibular or maxillary posterior teeth without any carious lesion or incipient carious lesion limited to the enamel, denoted as code 01, 02, and 03 according to the ICDAS II scoring system, were included. The teeth samples were examined under 10x magnification individually. Teeth were excluded from the study if there was the presence of dental fluorosis, tetracycline staining or any sources of staining, hypoplasia, and dentinal exposure.

Before the remineralisation process, two trained and calibrated examiners (T.Y.N and M.S.H) performed the visual assessment of each sample using ICDAS II. Visual examination was using a dental operating light, a WHO probe, and a 3-way syringe to score each tooth according to the ICDAS II scoring system. Sharp explorers were not used during the visual diagnosis. TYN and MSH scored the teeth individually with no discussion regarding the scores to ensure blinding. Interexaminer reproducibility was assessed using kappa analysis. The third examiner (M.F.R) used the DDPen to score each sample afterwards. A triple air syringe was used for five seconds to dry out the enamel surface before using the DDPen. Before scoring, the device was calibrated according to manufacturer's instructions. The probe tip was positioned on the occlusal surface of the tooth and rotated around its vertical axis until the highest value was found. ICDAS II scores and DDPen readings were repeated for two weeks for all samples to ascertain and evaluate intraexaminer reproducibility.

For the control group, the extracted teeth samples were washed with deionised water and then placed in artificial saliva at 37°C. No material was used for remineralisation of the control group. For the CPP-ACP with fluoride (GC Tooth Mousse Plus®) group, the paste was applied once daily (every 24 h) for 21 days on the occlusal surfaces with a microbrush tip applicator left in place for three minutes which simulated at-home use of this cream. The specimens were then rinsed with deionised water and gently dried with an air syringe. The samples were then immersed in artificial saliva and incubated at 37°C [26]. This process was repeated for 21 days. For the fluoride varnish (Colgate Duraphat®) group, a thin layer of the Duraphat® was applied once using a microbrush tip applicator on tooth's enamel surface, which simulated the professional application in a dental visit. The samples were then immersed in artificial saliva at 37°C for 6 h [27] to simulate the oral environment. Then, the varnish was carefully removed using a toothbrush [27, 28] to ensure the complete elimination of the surface layer of varnish [6, 29]. The samples were then rinsed for one minute with deionised water. After that, the samples were immersed in artificial saliva and incubated at 37°C.

After the remineralisation process, MFR repeats the scoring using the DDPen post-remineralisation for each sample. To ensure blinding, the examiner did not have access to previous scores of ICDAS or DDPen, and the same procedure for performing the post-remineralisation ICDAS score was followed. The teeth were then fixed in dental stone blocks and sectioned longitudinally using a diamond-coated bandsaw (Exakt system) for histological examination using a stereomicroscope with 10x magnification. Parallel cutting of the samples was performed by using tweezer prongs. The parallelism of the block is of utmost importance to ensure accurate cutting direction. The teeth sections fixed in the blocks were then polished by using slurry pumice. The polished sections were then again examined at 10x magnification under the stereomicroscope. Each section was photographed using a digital camera. This gold standard histological examination was compared with the ICDAS II scoring and DDPen examination for validity assessment.

The descriptive statistical analysis was performed using SPSS software version 24 (IBM SPSS Statistics). Inter- and intraexaminer reproducibility was assessed by calculating the unweighted kappa coefficient [30]. Wilcoxon signed ranks for pre-and post-remineralisation using different caries diagnostic tests were performed to assess the potential to detect remineralisation. Spearman's rank correlation of ICDAS-II and DDPen with the histological gold standard was performed to check the association. Sensitivity, specificity, and area under the ROC were calculated for ICDAS II and DDPen scores to compare both caries diagnostic methods.

## 3. Results

Kappa statistics revealed almost perfect reproducibility of TYN (0.954), MSH (0.919), and MFR (0.885), whereas good interexaminer agreement (0.673) was observed [31].  Table 1 describes the difference between the pre- and post-remineralisation scores, which were assessed using ICDAS-II and DDPen. According to ICDAS-II scores, a significant difference was noted in GC Tooth Mousse Plus® and Duraphat® study samples, whereas the difference between the pre- and post-remineralisation of the control group was not significant. According to the DDPen score criteria, a statistically significant difference was noted among all study groups; however, a greater significance level was noted in the GC Tooth Mousse Plus® and Duraphat® study samples compared with the control group.  Table 2 presents kappa values of the DDPen score with different cut-off values. The DDPen was noted to have the highest agreement compared with the histological gold standard described by Lussi et al. [32].  Table 3 presents the frequency of true-positive and false-positive findings of the carious occlusal lesion. The Spearman's rank correlation of ICDAS-II and DDPen with the histological gold standard revealed a higher association of DDPen score (.738) than ICDAS-II scores (.430).

**Figure 1:**
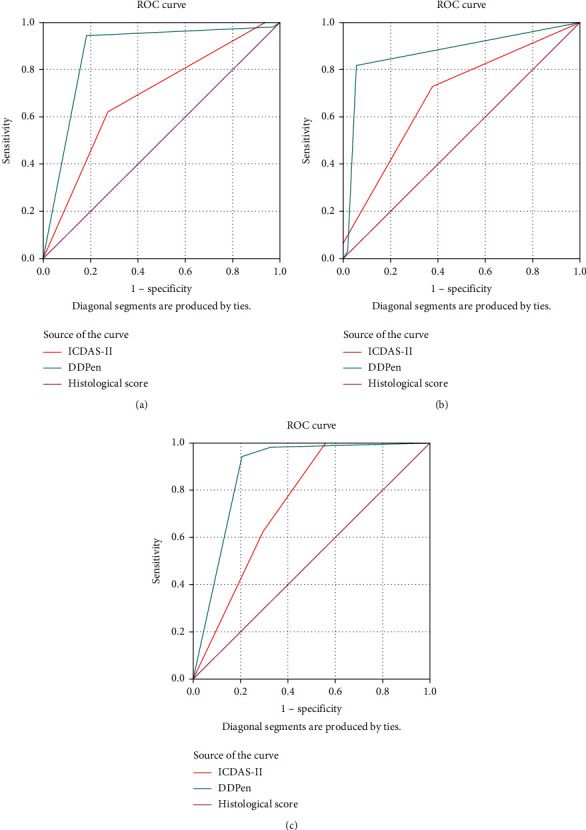
Area under the receiver-operating characteristic (AUROC) for International Caries Detection and Assessment System (ICDAS) codes under binary groups correlated with Downer's histologic classification at (a) D1 threshold, (b) D2 threshold, and (c) D3 threshold.

**Figure 2:**
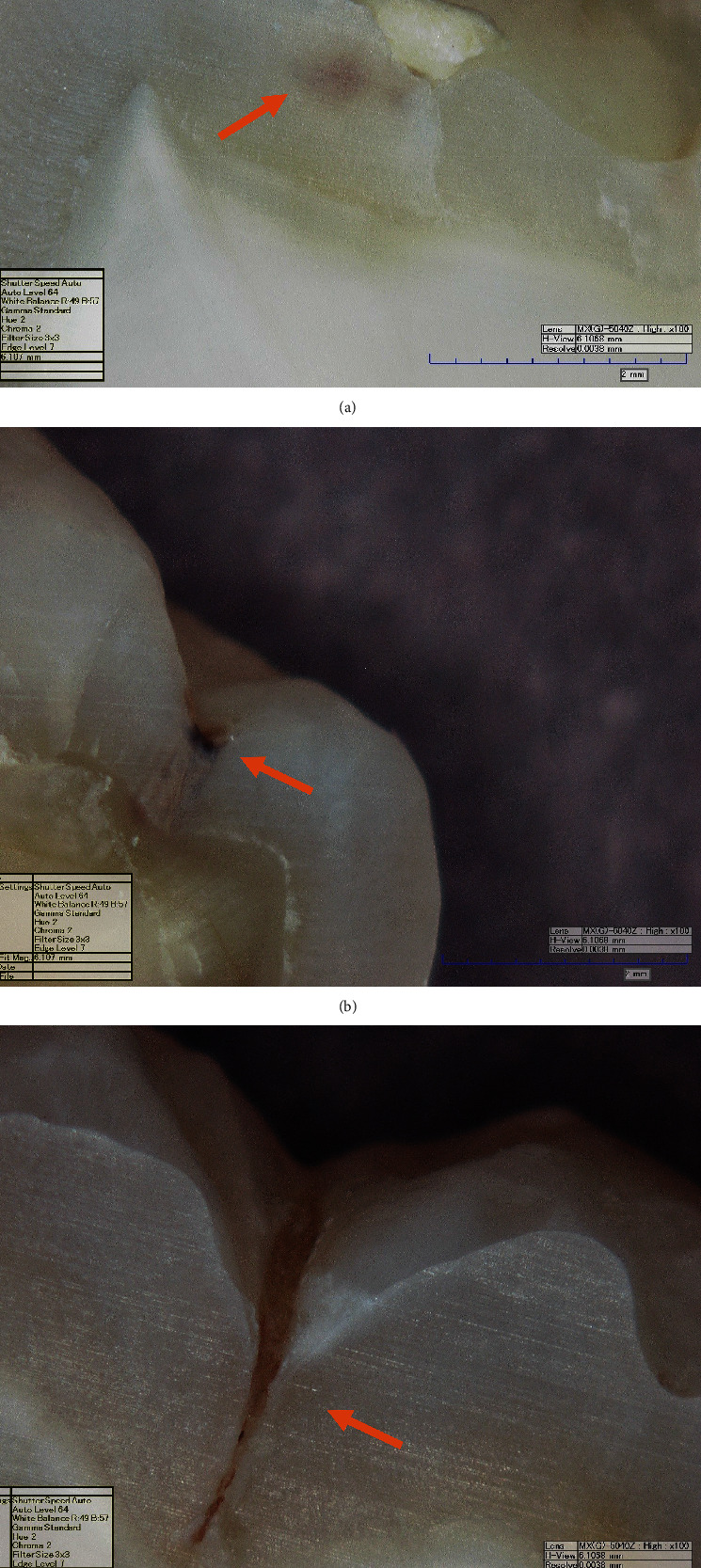
Histological section from Hirox digital microscope (KH-7700) (100x magnification) of tooth showing lesion at Downer's classification level (a) D1, (b) D2, and (c) D3.

Figure 1(a) presents the ROC curves plotted for the ICDAS-II code 0 and DDPen score (0-13) measurements compared with Downer's histological level DI extent as a gold standard validation.  Table 4 presents the *Az* value of DDPen score (0-13) at D1 (0.972) was higher than ICDAS-II code 0 at D1 (0.811), showing the greater accuracy of the method. Rank correlations (Spearman's coefficient) with histology D1 were .692 for ICDAS-II code 0 and .950 for DDPen score (0-13).  Figure 2(a) represents the histological section of the tooth at Downer's classification level D1.

Figure 1(b) presents the ROC curves plotted for the ICDAS-II codes 1 and 2 and DDPen score (14-20) measurements using Downer's histological level D2 extent as a gold standard validation.  Table 4 presents the *Az* value of DDPen score (14-20) at D2 (0.894) was higher than ICDAS-II codes 1 and 2 at D2 (0.667), showing the greater accuracy of the method. Rank correlations (Spearman's coefficient) with histology D2 were .769 for ICDAS-II codes 1 and 2 and .854 for DDPen score (14-20).  Figure 2(b) represents the histological section of a tooth at Downer's classification level D2.

Figure 1(c) presents the ROC curves plotted for the ICDAS-II code 3 and DDPen score (>21) measurements using Downer's histological level D3 extent as a gold standard validation.  Table 4 presents the *Az* value of DDPen score (>21) at D3 (0.838) was higher than ICDAS-II code 3 at D3 (0.721), showing the greater accuracy of the method. Rank correlations (Spearman's coefficient) with histology D3 were .601 for ICDAS-II code 3 and .774 for DDPen score (>21).  Figure 2(c) represents the histological section of a tooth at Downer's classification level D3.

## 4. Discussion

The current study is aimed at evaluating the sensitivity and specificity of ICDAS II and DDPen to detect the remineralisation of early enamel caries lesion in extracted human permanent posterior teeth and compare with the gold standard histological examination to identify the most suitable cut-off values of DDPen for identification of remineralised carious lesions.

The current study utilised ICDAS-II and DDPen scoring, which are designed to enable the clinicians to diagnose the disease at the chairside, making the results of the current study more relatable to a clinical setup. The current study did not find a significant difference in the remineralisation potential of GC Tooth Mousse Plus® and Duraphat®, following previous literature [6]. However, the effect of GC Tooth Mousse Plus® on salivary constituents and pH value were not considered. Varying cut-off limits for DDPen have been proposed and adopted in the laboratory [33, 34] and clinical studies [32, 35, 36]. Heinrich-Weltzien et al. [37] conducted a systematic review assessing different cut-off values and concluded that the cut-off values might be selected based on the extent of the carious lesion, and different cut-off values were correlated with enamel and dentinal carious lesions.

The cut-off values for DDPen, which had the highest and a significant correlation (0.72^∗^) with Downer's histological scores for the corresponding study samples, agree with the cut-off value suggested by Lussi et al. [32]. A possible explanation of this close agreement could be the assessment of freshly extracted teeth and the preservation medium (artificial saliva). The use of storage solutions such as formalin, sodium azide, or thymol-based mediums has been reported to affect the DDPen measurements [34, 38]. The values of fluorescence emission intensity decrease with time of immersion in all storage solutions, except for artificial saliva and glutaraldehyde [39]. The storage solutions tend to remove the organic compounds from the enamel surface. It is important to note that distilled water does not remove the organic compounds after 30 days of immersion [39]. Immersion in 0.1% thymol solution has shown a decline compared to the laser fluorescence values [40]. The area under the ROC curve (AUROC) can be defined as a composite measure of accuracy. Metz [41] stated that AUROC is a more meaningful measure of the value of a diagnostic test than the overall accuracy, as it does not depend on the disease prevalence in the population. The “diagnostic test” in the current study, being either ICDAS-II or DDPen, is compared with the gold standard Downers histological classification and “the disease” being remineralisation of the carious lesion.

An advantage of the area under the ROC curve analysis is that it reflects the diagnostic performance more comprehensively than the sensitivity and specificity, determined by only one cut-off point. It also provides an overall validity of the methods [42, 43]. Diniz et al. [44] performed an in vivo evaluation of the DDPen utilising the area under the ROC curve and reported that the *Az* value at D1 (0.72), D2 (0.81), and D3 (0.93), whereas another clinical study by Heinrich-Weltzien et al. [37] reported higher *Az* values for D2 (0.90) and D3 (0.83). The current study reported the highest Az value of DDPen score (0-13) at D1 (0.972) followed by DDPen score (14-20) at D2 (0.894) and then DDPen score (>21) at D3 (0.838). The difference in the performance of DDPen can be attributed to the determination of cut-off values or the number of cases reported in each study. Our study included the most significant number of cases having carious lesions at the D1 level, which could better represent higher accuracy in that category.

Iranzo-Cortés et al. [45] conducted a study focusing on occlusal caries by comparing the performance of ICDAS II and DDPen. The study concluded that the adjunct use of DDPen for the diagnosis of carious lesions was advisable. The results showed that DDPen demonstrated higher sensitivity, and ICDAS-II demonstrated higher specificity, following the current study results. A balance between sensitivity and specificity of a diagnostic tool is desired to ensure correct diagnosis and effective treatment planning. The current study found higher sensitivity of DDPen, which can sometimes lead to excessive treatment, which might not be deemed necessary when evaluated clinically.

On the other hand, a higher specificity is desirable to ensure minimal false-positive cases, which was found true in the case of ICDAS II. A possible explanation of this finding is the subjective nature of ICDAS criteria, which provides clinicians with a specific demarcation of disease progression. Similarly, Akgul et al. [1] performed a diagnostic evaluation of incipient carious lesion using visual inspection (VI), DDPen, DIAGNOdent Camera, and alternating-current-impedance-spectroscopy technique (ACIST). The study concluded that either of the diagnostic devices, when used alone, are not sensitive enough to diagnose incipient carious lesions, and these devices must be used as an adjunct to the traditional visual inspection method. The current study results also warrant the use of DDPen as an adjunct to the ICDAS II scoring system as both methods have their benefits, which add up together.

The spearman rank correlation coefficient was calculated for different lesion depths based on Downer's histological classification. The rank score of DDPen ranged from 0.774 to 0.950 compared to the ICDAS-II rank score ranging from 0.601 to 0.769. However, these results revealed the potential of ICDAS-II and DDPen to detect remineralisation. Another notable finding is that the most significant difficulty in diagnosing carious lesions was observed in deeper carious lesions with the lowest accuracy. The results revealed that DDPen could be utilised as a valuable clinical tool to detect remineralisation and monitor the progress of remineralisation of a particular lesion.

Alomari et al. [46] carried out a laboratory study evaluating the effectiveness by measuring the sensitivity and specificity of visual-only, visual + radiography, visual + radiography + DDPen for diagnosing noncavitated occluso-dentinal caries. The study concluded that although the use of radiography and DDPen adjunctively had a benefit, the *Az* value did not present a statistically significant difference, meaning that the diagnosis and decision-making were not influenced in the three study groups. In comparison, our study focused on carious lesions and found that DDPen was significantly (rank correlation = 0.738) more effective in diagnosing cavitated carious lesions when compared with ICDAS-II (rank correlation = 0.430), which warrants the use of DDPen in cavitated lesions as an adjunct. The higher accuracy of DDPen and electronic caries monitor has also been previously reported [47]. A possible explanation of this difference might be the colour of the lesion, which can mask the true nature of a lesion, hence, making it difficult to diagnose using only visual examination (ICDAS-II). The DDPen uses fluorescence which makes the diagnosis of a lesion effortless; however, it can also lead to an under-or over-estimation. Another explanation is the subjective nature of ICDAS-II, which is not so in the case of DDPen, which uses a pinpoint location to quantify the extent of a lesion. Another notable finding is that the most significant difficulty in diagnosing carious lesions was observed in deeper carious lesions with the lowest accuracy. A possible explanation is the fewer samples in the D3 level. Different cut-off values can be tested to increase the specificity of DDPen at the dentine level. Lussi et al. [33] also found lower specificity of DDPen at dentine level; however, no explanation of this phenomenon was hypothesised. The depth of a carious lesion might influence the absorbance of the laser, influencing the fluorescence of deeper carious lesions.

A change in either pH or the presence of free radical ions can create a desired anticariogenic environment, as previously noted in the literature [48, 49]. Another explanation is the limitation of assessing the effect of GC Tooth Mousse Plus® just by focusing on remineralisation. Other assessable properties include surface microhardness, pH cycling model, and salivary chemical analysis. These properties might reveal a benefit of using GC Tooth Mousse Plus® over conventional fluoride varnish. Although the current study did not find a significant difference between GC Tooth Mousse Plus® and Duraphat® regarding the remineralisation of cavitated carious lesion, one of the CPP-ACP advantages is an extended periodic release of calcium and phosphate ions which pose an anticariogenic effect. However, the current study design did not consider the role of free radical ions or the long-term effect of CPP-ACP over a three- or six-month period, which has been explored previously in the literature [50]. Future studies can focus on a clinical study design that is relatable to real-life clinical practice. The parallel comparison of other diagnostic devices and the currently employed options would allow better comparability of sensitivity and specificity of diagnostic device options. Salivary constituents of pre- and post application of remineralising agents over more extended follow-up periods might reveal exciting findings that can contribute towards the prevention of the carious process.

## 5. Conclusion

The ICDAS-II scoring system and DDPen scoring were able to detect remineralisation. A cut-off value of 0-13 denotes sound tooth, 14-20 denotes enamel caries, and >21 denotes dentine caries. The DDPen scoring had a higher-ranked correlation with Downer's histological scoring as compared to ICDAS-II scoring. The area under the ROC curve revealed that DDPen scoring demonstrated higher sensitivity while ICDAS-II scoring demonstrated greater specificity to detect remineralisation.

## Figures and Tables

**Table 1:** Wilcoxon signed ranks for pre- and post-remineralisation using different caries diagnostic tests.

	*Z*	*p* value
ICDAS-II		
Control	-1.342	.180
Tooth Mousse Plus®	-3.285	.001^∗∗^
Duraphat®	-2.592	.010^∗^
DDPen score		
Control	-2.928	.003^∗∗^
Tooth Mousse Plus®	-4.379	<.001^∗∗^
Duraphat®	-4.411	<.001^∗∗^

**Table 2:** Kappa statistics for different cut-off values of DDPen score.

DDPen criteria	Histological gold standard				
	Sound	Demineralised enamel		Demineralised dentine	Kappa value
		Outer half	Inner half		
[32]	0-13	14-20		>21	.725^∗∗^
[44]	0-14	15-21		>22	.627
[35]	0-15	16-25		>25	.437
[33]	0-4	5-10	11-18	>18	.457

**Table 3:** Percentage of occlusal carious lesions detected correctly utilizing different caries diagnostic methods at different histological levels.

Caries diagnostic method	Histology score	Percentage of occlusal carious lesions detected correctly at different histological levels *n*%		*r*_*S*_ (SE)
		True positive-false positive/total	%	
ICDAS-II				
Sound (code 0)	D1	33-20/53	62.26	.430 (.084)
ICDAS (codes 1 and 2)	D2	11-22/33	33	
ICDAS (code 3)	D3	15-19/34	44.117	
DDPen score				
0-13	D1	50-3/53	94.33	.738(.062)
14-20	D2	26-7/33	78.78	
>21	D3	23-11/34	67.64	

%: percentage; *r*_*s*_: Spearman rank correlation; *D*: Downer's classification.

**Table 4:** Spearman's correlation coefficients, AUROC curve, specificity, and sensitivity for the mode ICDAS scores, DDPen when compared with the level D1, D2, and D3 Downer's histologic level.

	Histo score	*r*_*s*_	Standard error	*A*_*z*_ (95% CI)	Standard error	Sensitivity	Specificity
ICDAS II code 0	D1	.692	.053	.811 (.726-.897)	.044	.623	.284
DDPen cutoff 0-13	D1	.950	.028	.972 (.935-1.000)	.034	.943	.194
ICDAS II codes 1 and 2	D2	.769	.059	.667 (.557-.776)	.056	.667	.333
DDPen cutoff 14-20	D2	.854	.050	.894 (.810-.978)	.043	.788	.069
ICDAS II code 3	D3	.601	.067	.721 (.605-.837)	.059	.441	.023
DDPen cutoff >21	D3	.774	.058	.838 (.740-.937)	.050	.676	.023

*A*_*z*_: area under receiver-operator curve; CI: confidence interval; *D*: Downer's classification; *r*_*s*_: Spearman's coefficient; *S*_*n*_: sensitivity; *S*_*P*_: specificity.

## Data Availability

The data set used in the current study will be made available at the reasonable request.
